# The clinical-histologic and prognostic characteristics in patients with a second primary non-small-cell lung cancer after a lobectomy

**DOI:** 10.1093/icvts/ivad155

**Published:** 2023-09-15

**Authors:** Lei-Lei Wu, Rang-Rang Wang, Jia-Yi Qian, Yu’e Liu, Shang-Shang Ma, Ming-Jun Li, Long-Yan Xie, Zhi-Xin Li, Kun Li, Bing-Yong Sheng, Jun-Rong Ding, Dong Xie

**Affiliations:** Department of Thoracic Surgery, Shanghai Pulmonary Hospital, School of Medicine, Tongji University, Shanghai, 200433, P. R. China; Huadong Hospital Affiliated to Fudan University, Shanghai, 200040, P. R. China; Shanghai General Hospital, Shanghai Jiao Tong University School of Medicine, Shanghai, P. R. China; Department of Thoracic Surgery, Shanghai Pulmonary Hospital, School of Medicine, Tongji University, Shanghai, 200433, P. R. China; School of Medicine, Tongji University, Shanghai, 200092, P. R. China; School of Medicine, Tongji University, Shanghai, 200092, P. R. China; School of Medicine, Tongji University, Shanghai, 200092, P. R. China; School of Medicine, Tongji University, Shanghai, 200092, P. R. China; Department of Thoracic Surgery, Shanghai Pulmonary Hospital, School of Medicine, Tongji University, Shanghai, 200433, P. R. China; Department of Thoracic Surgery, Shanghai Pulmonary Hospital, School of Medicine, Tongji University, Shanghai, 200433, P. R. China; Radiology Department, Shanghai Pulmonary Hospital, School of Medicine, Tongji University, Shanghai, 200433, P. R. China; Department of Thoracic Surgery, Shanghai Pulmonary Hospital, School of Medicine, Tongji University, Shanghai, 200433, P. R. China; Department of Thoracic Surgery, Shanghai Pulmonary Hospital, School of Medicine, Tongji University, Shanghai, 200433, P. R. China

**Keywords:** Second primary lung cancer, propensity-score matching, histology migration, surgical treatment

## Abstract

**OBJECTIVES:**

The goal of this study was to investigate whether an operation can offer survival benefits for patients with a second primary non-small-cell lung cancer (NSCLC) after a lobectomy for a first primary NSCLC and to analyse the characteristics affecting the survival of those patients.

**METHODS:**

We performed survival analyses of patients with a second primary NSCLC based on the Surveillance, Epidemiology and End Results program and used propensity score matching to reduce the potential bias and analyse the data. In addition, the primary observational end point was overall survival (OS), and the secondary observational end point was histologic migration.

**RESULTS:**

The data from 944 patients were used to perform the main analysis. A total of 36.2% of patients experienced a shift in tumour histologic type between 2 diagnoses of primary NSCLC, and this shift significantly affected OS (*P* = 0.0065). The median survival time in patients with surgical resection and those without an operation was 52.0 months versus 33.0 months, respectively. Patients with surgical resection at the secondary diagnosis had better survival than those without surgery (5-year OS rate: 48.0% vs 34.0%, *P* < 0.001). In addition, compared with a pneumonectomy and a sublobar resection, a lobectomy was the optimal surgical procedure for patients diagnosed with a second primary NSCLC after adjusting for other confounders (adjusted hazard ratio: 0.68, *P* < 0.01). However, in the subgroup analysis, lobar and sublobar resections could provide similar survival benefits for patients with tumour size ≤20 mm (*P* = 0.5).

**CONCLUSIONS:**

The operation, especially a lobectomy, can prolong OS in patients with a second primary NSCLC. Besides, sublobar resection can be performed in selected patients with tumour size ≤20 mm. Moreover, histologic migration may impact the survival of those patients with a secondary primary NSCLC.

**Figure:**
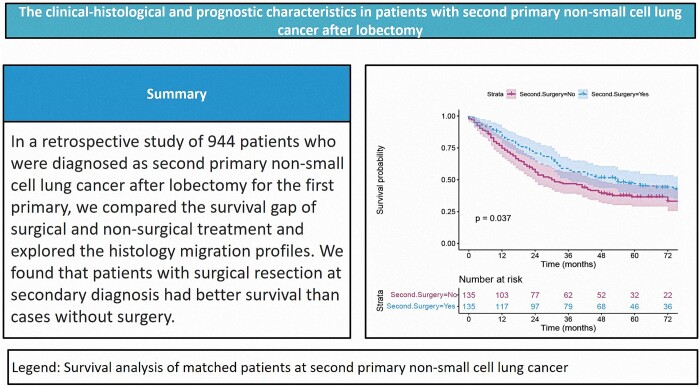

## INTRODUCTION

Lung cancer has become the second most commonly diagnosed type of cancer after female breast cancer but remains the leading cause of cancer deaths [1]. Thanks to biomarker-driven treatments, screening of high-risk populations and advances in drug development, the 5-year survival rate of lung cancer patients has increased from 17.2% 10 years ago to 22.9% now [2–4]. In addition, the advancement of minimally invasive surgery has further reduced the perioperative mortality rate of patients and of postoperative complications [5]. Encouragingly, the 5-year survival rate of patients with operable non-small-cell lung cancer (NSCLC) ranges from 52% to 94.3% [6, 7], with deference in survival rate influenced by varied tumour stages and healthcare disparities.

Unfortunately, some patients who underwent surgical resection at the first diagnosis still suffer torment from a second primary NSCLC, ranging from about 5.4% to 18.3% [7, 8]. However, whether patients with a second primary NSCLC can, after surgery, benefit from the second operation and the optimal surgical approach remains unclear.

To explore whether patients with a second primary NSCLC can benefit from a second surgical resection and explore the optimal surgical approach, we used the Surveillance, Epidemiology, and End Results (SEER) program to analyse the overall survival (OS) of patients with a second primary NSCLC. Meanwhile, we rematched the data by propensity score matching (PSM) methods to reduce the interference of data bias and confounding variables for further analysis.

## PATIENTS AND METHODS

### Ethics statement

The ethics committee of the Shanghai Pulmonary Hospital approved this study (No. K22-209), and patient informed consent was waived because of the anonymity of the data. The human data were handled in the manuscript in accordance with the Declaration of Helsinki.

### Data source and retrieval criteria

**Figure 1:**
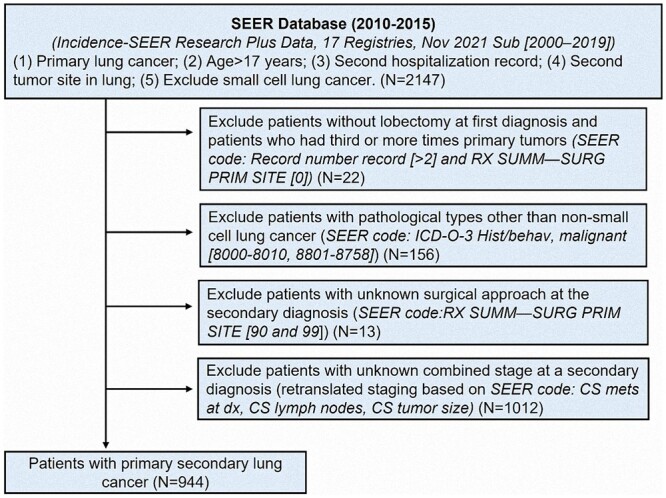
Flow chart for this study.

The data of patients who participated in this study were obtained from on the SEER database [SEER*Stat software, version 8.4.0 (Incidence-SEER Research Plus Data, 17 Registries, Nov 2021 Sub 2000–2019)]. We retrieved the relevant clinical information of patients registered in the SEER database from 2010 to 2015. The retrieval criteria included patients with 2 diagnoses of primary lung cancer (excluding small-cell lung cancer) and older than 17 years. Detailed information about the SEER code and the use code for patient selection is presented in Supplementary Table 1. The exclusion criteria for this study are shown in Fig. 1. Finally, we accepted 944 patients who met the requirements and had been diagnosed twice with primary NSCLC.

### Statistical analyses

Categorical variables were analysed using the χ^2^ test. Given the limited information in the SEER database, we dealt with the missing information by exclusion. The Kaplan-Meier method was used to draw the survival curves, which were compared using the log-rank test [9]. *P*-values between subgroups were compared using the Benjamini & Hochberg method. Propensity score matching was used to reduce the interference caused by data bias and confounding factors [10, 11]. We matched and balanced the clinical information of patients who received and did not receive the second operation by using the nearest neighbour method without a replacement method with 1:1 matching. The caliper value was set to 0.05 the logit of the propensity score. We included sex, race, tumour location, age, histologic analysis, surgery, radiotherapy, chemotherapy, marital status, grade, laterality and tumour-node-metastasis (TNM) stage to perform a PSM. The standardized mean difference was used to evaluate the balance effect of PSM. Its threshold was 0.2, below which the match was considered to be balanced. The independent risk factors that affect the patient's prognosis and the corresponding hazard ratio (HR) and 95% confidence interval (CI) values were calculated using the Cox regression model (clustered standard errors). The primary observational end point was overall survival (OS), and the secondary observational end point was histologic migration. The time interval between the diagnosis of the secondary primary NSCLC and death was defined as the OS. *P* < 0.05 was considered statistically significant. All statistical analyses in this study were performed using the software from R Studio version 4.0.1 (https://www.rstudio.com/).

### Follow-up

The follow-up information from the SEER database was complete. Therefore, those patients had definitive survival statuses, including dead and alive. Because detailed information on follow-up in the SEER database is not clear, we recommend that patients visit the outpatient clinic at 3- or 6-month intervals postoperatively for the first 3 years and at 12-month intervals after that. In addition, we used telephone and outpatient visit records for follow-up updates.

## RESULTS

### Patient populations

A total of 944 patients who were diagnosed twice with primary NSCLC were included and were divided into 2 groups based on whether they were operated on a second time. Among them, 419 (44.4%) were males, and 525 (55.6%) were females. All 944 patients received a lobectomy when they were diagnosed for the first time; 615 patients received surgical treatment when they were diagnosed for the second time; and 329 patients did not receive surgical treatment. The clinical characteristics of patients at the first diagnosis and secondary diagnosis are shown in Supplementary Table 2 and Table 1.

**Table 1:** The characteristics of patients with a second non-small-cell lung cancer

Variable			Secondary surgery		
		Overall	No	Yes	*P*-value
		N = 944	N = 329	N = 615	
Sex (%)	Female	525 (55.6)	176 (53.5)	349 (56.7)	0.374
	Male	419 (44.4)	153 (46.5)	266 (43.3)	
Race (%)	Other	151 (16.0)	57 (17.3)	94 (15.3)	0.47
	White	793 (84.0)	272 (82.7)	521 (84.7)	
Age (%)	<65 years	668 (70.8)	247 (75.1)	421 (68.5)	0.04
	≥65 years	276 (29.2)	82 (24.9)	194 (31.5)	
Marital status (%)	Married	507 (53.7)	180 (54.7)	327 (53.2)	0.453
	Unmarried	372 (39.4)	131 (39.8)	241 (39.2)	
	Unknown	65 (6.9)	18 (5.5)	47 (7.6)	
Location (%)	Upper lobe	485 (51.4)	167 (50.8)	318 (51.7)	<0.001
	Middle lobe	69 (7.3)	17 (5.2)	52 (8.5)	
	Lower lobe	358 (37.9)	119 (36.2)	239 (38.9)	
	Other/unknown	32 (3.4)	26 (7.9)	6 (1.0)	
Histologic analysis (%)	ADC	609 (64.5)	207 (62.9)	402 (65.4)	0.001
	SCC	180 (19.1)	81 (24.6)	99 (16.1)	
	Unknown or other NSCLC	155 (16.4)	41 (12.5)	114 (18.5)	
Radiotherapy (%)	No	696 (73.7)	132 (40.1)	564 (91.7)	<0.001
	Yes	244 (25.8)	195 (59.3)	49 (8.0)	
	Unknown	4 (0.4)	2 (0.6)	2 (0.3)	
Chemotherapy (%)	No	707 (74.9)	214 (65.0)	493 (80.2)	<0.001
	Yes	237 (25.1)	115 (35.0)	122 (19.8)	
Grade (%)	I	179 (19.0)	45 (13.7)	134 (21.8)	<0.001
	II	288 (30.5)	54 (16.4)	234 (38.0)	
	III	181 (19.2)	45 (13.7)	136 (22.1)	
	IV	11 (1.2)	4 (1.2)	7 (1.1)	
	Unknown	285 (30.2)	181 (55.0)	104 (16.9)	
Laterality (%)	Left	420 (44.5)	154 (46.8)	266 (43.3)	0.001
	Right	517 (54.8)	168 (51.1)	349 (56.7)	
	Other/unknown	7 (0.7)	7 (2.1)	0 (0.0)	
Surgery (%)	No	329 (34.9)	329 (100.0)	0 (0.0)	<0.001
	Lobar resection	301 (31.9)	0 (0.0)	301 (48.9)	
	Pneumonectomy	13 (1.4)	0 (0.0)	13 (2.1)	
	Sublobar resection	301 (31.9)	0 (0.0)	301 (48.9)	
TNM stage (%)	I	691 (73.2)	189 (57.4)	502 (81.6)	<0.001
	II	93 (9.9)	28 (8.5)	65 (10.6)	
	IIIA	51 (5.4)	26 (7.9)	25 (4.1)	
	IIIB	15 (1.6)	12 (3.6)	3 (0.5)	
	IV	94 (10.0)	74 (22.5)	20 (3.3)	

ADC: adenocarcinoma; NSCLC: non-small-cell lung cancer; SCC: squamous cell carcinoma; TNM: tumour-node-metastasis.

### Survival analysis

**Figure 2:**
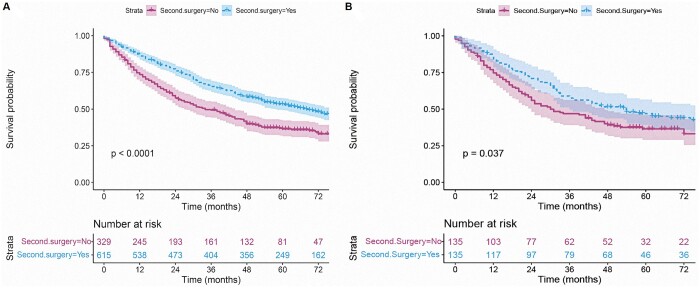
Survival analysis of patients with a second primary non-small-cell lung cancer before (**A**) and after (**B**) propensity score matching (the adjusted *P* values between subgroups were analysed using the Benjamini & Hochberg method).

**Figure 3:**
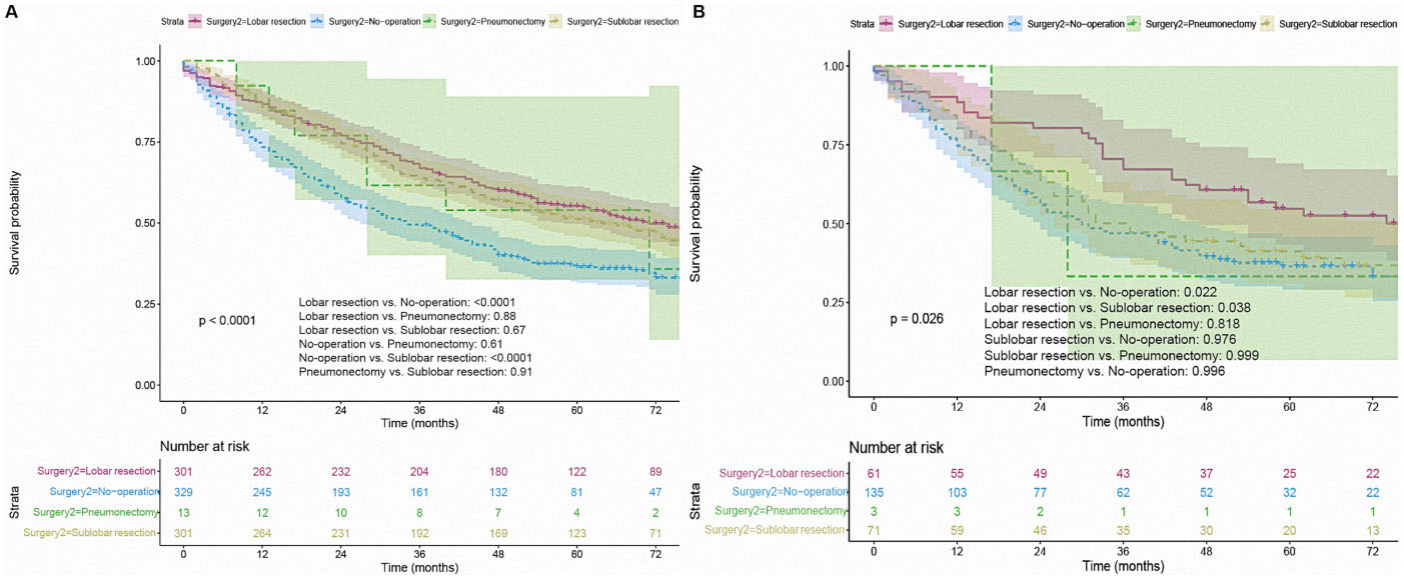
Overall survival analysis of different surgical approaches in unmatched (**A**) and matched (**B**) cohorts.

**Figure 4:**
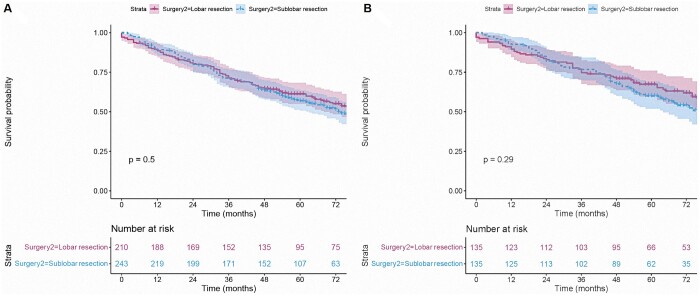
Survival analysis of patients with tumour size ≤20 mm before (**A**) and after (**B**) matching.

For patients with a second primary NSCLC, the findings of the Kaplan-Meyer analysis revealed that those who underwent surgery for the second diagnosed primary NSCLC had a better prognosis in terms of OS than those who did not (unadjusted HR = 0.62, 95% CI 0.52–0.74, *P *<* *0.001, Fig. 2A). Patients with surgical resection at the secondary diagnosis had better survival than patients without surgery (*P* < 0.001). After PSM, 135 patients were drawn from each group (surgery vs no surgery). There was no statistical difference in demographic data and related clinical features between the 2 groups (all standardized mean difference ≤ 0.2; Supplementary Table 3 and Supplementary Fig. 1). Similarly, patients who were operated on had a more improved OS than those who were not operated on in the matched cohort (PSM-adjusted HR = 0.73, *P* = 0.037, Fig. 2B). Moreover, both sublobar and lobar resection provided survival benefits (all *P* < 0.001, Table 2 and Fig. 3A). In the matched cohort, patients only got survival benefits from a lobectomy (PSM-adjusted HR = 0.535, *P* = 0.022, Fig. 3B). Accordingly, a subgroup analysis was performed in the cohort with tumour size ≤20 mm. The results indicated that a lobectomy and a sublobar resection had similar effects on the prognosis of patients with a second primary NSCLC after a lobectomy for first primary NSCLC (before matching: *P* = 0.5, Fig. 4A; after matching: *P* = 0.29).

**Table 2:** Univariable and multivariable Cox regression analyses of variables in the secondary diagnosis for overall survival

Characteristic	Univariable analysis			Multivariable analysis		
	HR	95% CI	*P*-value	HR	95% CI	*P*-value
Sex						
Female	Ref			Ref		
Male	1.52	1.28-1.80	<0.001	1.40	1.20-1.70	<0.001
Race						
Other	Ref					
White	1.15	0.91-1.46	0.247			
Location						
Lower lobe	Ref			Ref		
Upper lobe	0.84	0.70-1.01	0.057	0.78	0.65-0.94	0.008
Middle lobe	0.99	0.71-1.38	0.949	0.92	0.65-1.30	0.64
Other/unknown	1.75	1.15-2.68	0.009	1.4	0.82-2.2	0.23
Histologic analysis						
ADC	Ref			Ref		
SCC	1.54	1.25-1.9	<0.001	1.20	0.95-1.5	0.13
Unknown or other NSCLC	1.06	0.84-1.34	0.606	1.10	0.84-1.40	0.52
Surgery						
No	Ref			Ref		
Sublobar resection	0.64	0.52-0.79	<0.001	0.89	0.70-1.10	0.32
Lobar resection	0.59	0.48-0.73	<0.001	0.68	0.53-0.86	0.0012
Pneumonectomy	0.67	0.32-1.42	0.295	0.82	0.38-1.80	0.62
Radiotherapy						
No	Ref					
Yes	1.14	0.95-1.38	0.164			
Unknown	0.86	0.22-3.46	0.834			
Chemotherapy						
No	Ref			Ref		
Yes	1.48	1.23-1.78	<0.001	0.99	0.80-1.20	0.92
Marital status						
Married	Ref					
Unmarried	1.10	0.92-1.31	0.285			
Unknown	0.80	0.55-1.16	0.246			
Grade						
I	Ref			Ref		
II	1.25	0.96-1.63	0.094	1.0	0.76-1.30	0.98
III	1.71	1.29-2.26	<0.001	1.2	0.86-1.60	0.34
IV	4.96	2.64-9.32	<0.001	2.2	1.10-4.50	0.025
Unknown	1.48	1.14-1.92	0.003	0.97	0.73-1.30	0.81
Age						
≥65 years	Ref			Ref		
<65 years	0.65	0.53-0.79	<0.001	0.63	0.51-0.77	<0.001
Laterality						
Left	Ref					
Right	0.93	0.78-1.1	0.385			
TNM stage						
I	Ref			Ref		
II	2.43	1.88-3.14	<0.001	2.40	1.80-3.20	<0.001
IIIA	2.08	1.48-2.93	<0.001	1.90	1.30-2.70	<0.001
IIIB	3.78	2.21-6.46	<0.001	4.20	2.40-7.40	<0.001
IV	4.11	3.22-5.24	<0.001	3.60	2.80-4.80	<0.001

ADC: adenocarcinoma; NSCLC: non-small-cell lung cancer; SCC: squamous cell carcinoma; TNM: tumour-node-metastasis.

### Univariable and multivariable Cox regression analyses for the unmatched cohort

To clarify the independent factor affecting the OS of patients with a second primary NSCLC, we first performed a univariable Cox regression analysis of all variables and then performed a multivariable Cox regression analysis based on the results of univariable Cox regression analysis [12] (Table 2). For patients with a second primary NSCLC, lobar resection was an independent factor with a good prognosis (adjusted HR = 0.68; 95% CI 0.53–0.86; *P* = 0.0012). In addition, when diagnosed with a second primary NSCLC, patients with a squamous cell carcinoma (SCC) had a worse prognosis (unadjusted HR = 1.54, 95% CI 1.25–1.90, *P* < 0.001). However, SCC could not be an independent prognostic indicator after adjusting for other confounders. Overall, sex, tumour location, lobar resection, grade, age and tumour-node-metastasis (TNM) stage were confirmed as independent prognostic indicators (all *P* < 0.05, Table 2**)**.

### Histologic migration: exploratory discovery

**Figure 5:**
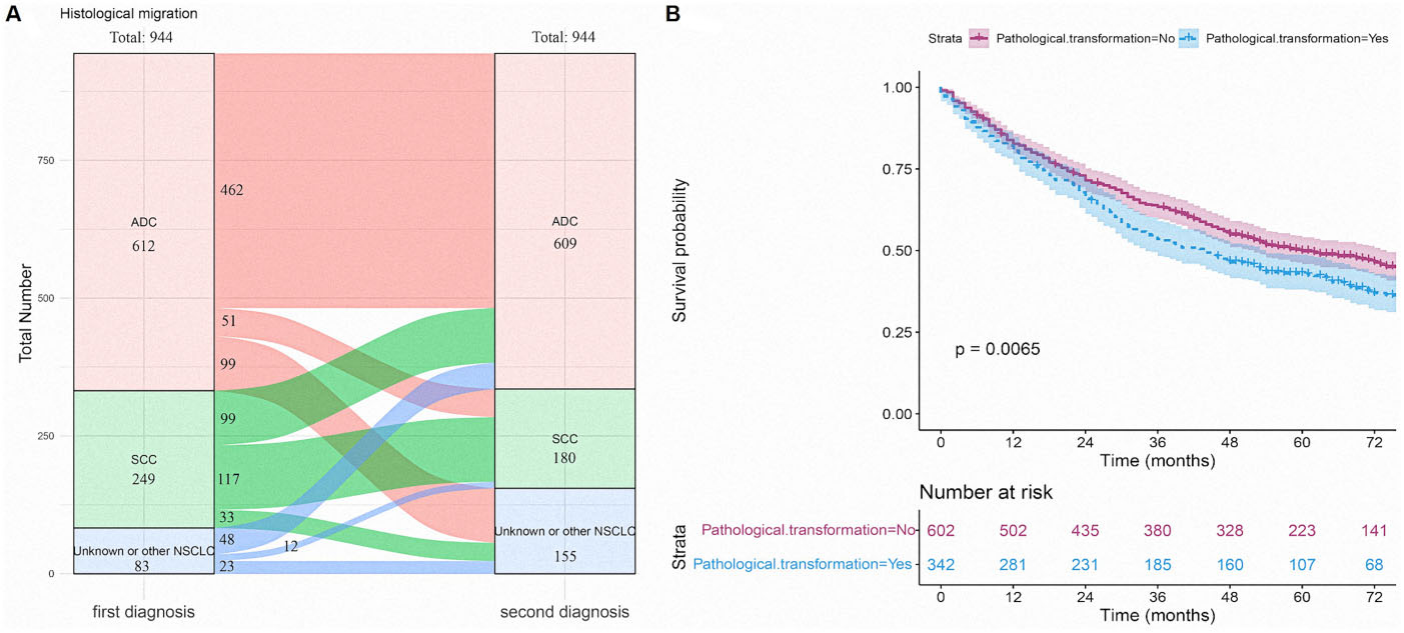
(**A**) Sankey diagram of histologic migration; (**B**) Survival analysis of patients with pathological transformation or without non-pathological transformation. ADC: adenocarcinoma; NSCLC: non-small-cell lung cancer. SCC: squamous cell carcinoma

Histologic migration in patients who had 2 diagnoses of primary NSCLC is shown in the Sankey diagram (Fig. 5A). The results demonstrated that the overall histologic types of cancers in patients who received 2 diagnoses were similar. Among the patients diagnosed with primary NSCLC for the first time, 612 (64.8%) had adenocarcinomas (ADC) and 166 (26.3%) had SCC. For those who received a second diagnosis, 441 (64.5%) had ADC, and 187 (19.1%) had SCCs. Compared with their first diagnosis of ADC, 462 (75.5%) patients received a second diagnosis of ADC, and 51 (8.3%) migrated to SCC. For patients whose first diagnosis was SCC, 117 (47.0%) had a second diagnosis of SCC, and 99 (39.8%) migrated to ADC (Fig. 5A). Overall, 342 (36.2%) patients underwent a shift in histologic type. Moreover, a patient's histologic migration during the 2 diagnoses significantly affected the patient's OS (*P* = 0.0065; Fig. 5B).

## DISCUSSION

Surgical resection is the preferred treatment method for patients with early primary lung cancer [13, 14], and the standard surgical method recommended by current guidelines is lobectomy [13, 15]. However, whether patients with a second diagnosis of primary lung cancer should receive surgical treatment is still controversial, and the recommended standard surgical approach has not yet been determined [16, 17]. Our research showed the following: (1) For patients with primary lung cancer who received surgical treatment for the first time, surgical treatment could still provide significant benefits to those patients when primary lung cancer was diagnosed for the second time; (2) lobar resection was worth considering for patients diagnosed with a second primary NSCLC; (3) the overall proportion of histologic types among patients with 2 diagnoses of primary lung cancer is similar; however, the component ratio of pathological types might not be same. Importantly, pathological transformation during 2 diagnoses significantly affected the OS of patients with a second primary NSCLC.

Most thoracic surgeons believe that surgical resection is the most valuable treatment for patients with a second primary lung cancer, but the degree of resection is still not unanimously recognized [18, 19]. It is mainly related to the patient's age, lung function and tumour classification when he or she was diagnosed for the second time [20, 21]. However, our study found that age is not a risk factor affecting the survival of patients with a second primary NSCLC. The survival time of patients receiving surgical treatment is significantly longer than that of patients who do not receive surgical treatment. Asaph *et al.* also believe that surgical treatment is beneficial for patients diagnosed with primary lung cancer for the second time [22]. Although the operation increases the risks associated with the patient's perioperative period, there is no obvious evidence that an operation should be banned [23].

Abid *et al.* think that sublobar resection is a more compromised surgical approach for patients diagnosed with primary lung cancer for the second time [21]. However, our research showed that patients who underwent lobotomy get the best benefits from that treatment. On the contrary, compared with patients who did not undergo surgery, the benefits of sublobar resection for patients were not obvious. It is undeniable that the second operation to remove the lobes of the lungs will further reduce the patient's residual lung function [18, 23]; however, this is not the main excuse for the compromise on the surgical method. Moreover, lobectomy is still the surgical approach that can bring more benefits to the survival of patients than other surgical methods, according to a previous study [24].

Our study also suggested that a lobectomy could prolong the prognosis for patients with a second primary tumour. Recently, clinical trials of GCOG0802 and CALGB140503 confirmed that a sublobar resection was not inferior to a lobectomy with respect to prognosis in patients with peripheral NSCLC with a tumour size of 2 cm or less and N0 disease [7, 25]. Those results provided the clinical guide to selecting a surgical approach for NSCLC patients with a tumour size of 2 cm or less and N0 disease. Besides, the sublobar resection could protect more lung function than the lobectomy. Thus, in the present study, we performed a subgroup analysis in patients with tumour size ≤20 mm, and found that a lobectomy and a sublobar resection had a similar effect on the survival of those patients. However, the proportion of patients with tumour size ≥20 mm was more than 30% in the present study, indicating that more than 30% of patients might not be suitable to undergo a sublobar resection. A lobectomy allows for a true anatomical resection by taking fuller account of tumour margins and vascular and lymphatic drainage issues [26]. Thus, a lobectomy is more advantageous in patients with more advanced stages or in terms of long-term patient survival [27]. Thoracic surgery colleagues should evaluate the patient's age, preoperative pulmonary function and clinical TNM staging before a second operation [28]. Moreover, the patient's lung function could be further improved through rehabilitation exercises [29]. Therefore, after assessing the patient's lung function and the risk of a secondary procedure, the operation may be performed for better survival benefit: A lobectomy is worth considering.

Minimal attention was paid to the migration of histologic types in patients with twice-diagnosed primary NSCLC. We found that the pathological types of patients were generally similar during the 2 diagnoses and that transformation occurred to some degree. Moreover, this histologic migration significantly impacted the OS of patients. A previous study reported that the consistency of the histologic type during the second diagnosis of primary lung cancer is an important prognostic factor for the second primary lung cancer [30]. Our findings were similar to theirs; however, we still need more evidence to prove this point.

Our research indicated that the second operation can still provide survival benefits for patients with primary lung cancer and that lobar resection is worth considering for patients diagnosed with primary NSCLC for the second time. We also found that for patients with 2 diagnoses of primary lung cancer, the pathological type was not necessarily consistent during the diagnosis and that this migration in histologic type had a significant effect on the OS of patients.

The present study still has some shortcomings. First, the study was retrospective; therefore, selection bias was not inevitable. Second, the number of cases after PSM was small. The small sample may affect the reliability of the conclusions. Third, since the SEER database does not have indicators such as lung function, comparing patients' lung function before a secondary surgery is impossible; thus, determining whether patients refused surgery because of poor lung function is difficult. Finally, we need more studies to confirm our findings.

## CONCLUSIONS

The operation, especially a lobectomy, can prolong OS in patients with a second primary NSCLC. In addition, sublobar resection can be performed in selected patients with tumour size ≤20 mm. Moreover, histologic migration may impact the survival of those patients with a secondary primary NSCLC. More prospective clinical trials are needed to verify the findings of our study.

## Supplementary Material

ivad155_Supplementary_Data

## Data Availability

All data for patients who participated in this study were obtained from the SEER database (https://seer.cancer.gov/), which is a public database. The data sets are available from the corresponding author upon reasonable request.

## Abbreviations

ADC: Adenocarcinoma
CI: Confidence interval
HR: Hazard ratio
NSCLC: Non-small-cell lung cancer
OS: Overall survival
PSM: Propensity score matching
SCC: Squamous cell carcinoma
SEER: Surveillance, Epidemiology, and End Results
