# Supplementary material for: The clinical-histologic and prognostic characteristics in patients with a second primary non-small-cell lung cancer after a lobectomy
Source: Interdiscip Cardiovasc Thorac Surg. 2023 Sep 15;37(3):ivad155. doi: 10.1093/icvts/ivad155 (PMC10521628; doi:10.1093/icvts/ivad155)
Supplement: ivad155_Supplementary_Data [file ivad155_supplementary_data.zip › Supplementary Table 3.docx]

**Supplementary Table 2.** The characteristics of patients at a second non-small cell lung cancer after propensity-score matching.

| **Variable** | |  | **Surgery** | |  |
| --- | --- | --- | --- | --- | --- |
|  |  | **Overall** | **No** | **Yes** | **p-value** |
|  |  | N=270 | N=135 | N=135 |  |
| Sex (%) | Male | 124 (45.9) | 64 (47.4) | 60 (44.4) | 0.714 |
|  | Female | 146 (54.1) | 71 (52.6) | 75 (55.6) |  |
| Race (%) | White | 230 (85.2) | 116 (85.9) | 114 (84.4) | 0.864 |
|  | Other | 40 (14.8) | 19 (14.1) | 21 (15.6) |  |
| Location (%) | Upper Lobe | 130 (48.1) | 68 (50.4) | 62 (45.9) | 0.47 |
|  | Middle Lobe | 16 (5.9) | 5 (3.7) | 11 (8.1) |  |
|  | Lower Lobe | 118 (43.7) | 59 (43.7) | 59 (43.7) |  |
|  | Other/Unknown | 6 (2.2) | 3 (2.2) | 3 (2.2) |  |
| Histology (%) | ADC | 175 (64.8) | 87 (64.4) | 88 (65.2) | 0.527 |
|  | SCC | 52 (19.3) | 29 (21.5) | 23 (17.0) |  |
|  | Unknown or other NSCLC | 43 (15.9) | 19 (14.1) | 24 (17.8) |  |
| Radiotherapy (%) | No | 186 (68.9) | 94 (69.6) | 92 (68.1) | 0.965 |
|  | Yes | 82 (30.4) | 40 (29.6) | 42 (31.1) |  |
|  | Unknown | 2 (0.7) | 1 (0.7) | 1 (0.7) |  |
| Chemotherapy (%) | No | 192 (71.1) | 93 (68.9) | 99 (73.3) | 0.502 |
|  | Yes | 78 (28.9) | 42 (31.1) | 36 (26.7) |  |
| Marital status (%) | Unmarried | 103 (38.1) | 50 (37.0) | 53 (39.3) | 0.827 |
|  | Married | 153 (56.7) | 77 (57.0) | 76 (56.3) |  |
|  | Unknown | 14 (5.2) | 8 (5.9) | 6 (4.4) |  |
| Grade (%) | I | 47 (17.4) | 23 (17.0) | 24 (17.8) | 0.811 |
|  | II | 64 (23.7) | 36 (26.7) | 28 (20.7) |  |
|  | III | 56 (20.7) | 28 (20.7) | 28 (20.7) |  |
|  | IV | 5 (1.9) | 2 (1.5) | 3 (2.2) |  |
|  | Unknown | 98 (36.3) | 46 (34.1) | 52 (38.5) |  |
| Age (%) | <65 years | 189 (70.0) | 94 (69.6) | 95 (70.4) | 1 |
|  | ≥65 years | 81 (30.0) | 41 (30.4) | 40 (29.6) |  |
| TNM stage (%) | 1 | 183 (67.8) | 88 (65.2) | 95 (70.4) | 0.844 |
|  | 2 | 23 (8.5) | 13 (9.6) | 10 (7.4) |  |
|  | 3 | 21 (7.8) | 11 (8.1) | 10 (7.4) |  |
|  | 4 | 6 (2.2) | 4 (3.0) | 2 (1.5) |  |
|  | 5 | 37 (13.7) | 19 (14.1) | 18 (13.3) |  |
| Laterality (%) | Right | 148 (54.8) | 67 (49.6) | 81 (60.0) | 0.112 |
|  | Left | 122 (45.2) | 68 (50.4) | 54 (40.0) |  |

ADC: adenocarcinoma; SCC: squamous cell carcinoma.
