# Supplementary material for: The clinical-histologic and prognostic characteristics in patients with a second primary non-small-cell lung cancer after a lobectomy
Source: Interdiscip Cardiovasc Thorac Surg. 2023 Sep 15;37(3):ivad155. doi: 10.1093/icvts/ivad155 (PMC10521628; doi:10.1093/icvts/ivad155)
Supplement: ivad155_Supplementary_Data [file ivad155_supplementary_data.zip › Supplementary Table 2.docx]

**Supplementary Table 2.** The characteristics of patients at a first non-small cell lung cancer

| **Variable** | |  | **Secondary surgery** | |  |
| --- | --- | --- | --- | --- | --- |
|  |  | **Overall** | **No** | **Yes** | **p-value** |
|  |  | **N=944** | **N=329** | **N=615** |  |
| Age (%) | <65 years | 319 (33.8) | 107 (32.5) | 212 (34.5) | 0.595 |
|  | ≥65 years | 625 (66.2) | 222 (67.5) | 403 (65.5) | |
| Marital status (%) | Married | 527 (55.8) | 189 (57.4) | 338 (55.0) | 0.764 |
|  | Unmarried | 372 (39.4) | 125 (38.0) | 247 (40.2) | |
|  | Unknown | 45 (4.8) | 15 (4.6) | 30 (4.9) | |
| Location (%) | Upper Lobe | 581 (61.5) | 209 (63.5) | 372 (60.5) | 0.088 |
|  | Middle Lobe | 48 (5.1) | 9 (2.7) | 39 (6.3) |  |
|  | Lower Lobe | 297 (31.5) | 103 (31.3) | 194 (31.5) |  |
|  | Other/Unknown | 18 (1.9) | 8 (2.4) | 10 (1.6) | |
| Histology (%) | ADC | 612 (64.8) | 209 (63.5) | 403 (65.5) | 0.342 |
|  | SCC | 249 (26.4) | 95 (28.9) | 154 (25.0) | |
|  | Unknown or other NSCLC | 83 (8.8) | 25 (7.6) | 58 (9.4) |  |
| Radiotherapy (%) | No | 851 (90.1) | 289 (87.8) | 562 (91.4) | 0.04 |
|  | Unknown | 4 (0.4) | 0 (0.0) | 4 (0.7) |  |
|  | Yes | 89 (9.4) | 40 (12.2) | 49 (8.0) |  |
| Chemotherapy (%) | No | 675 (71.5) | 230 (69.9) | 445 (72.4) | 0.472 |
|  | Yes | 269 (28.5) | 99 (30.1) | 170 (27.6) | |
| Grade (%) | I | 150 (15.9) | 44 (13.4) | 106 (17.2) | 0.149 |
|  | II | 414 (43.9) | 161 (48.9) | 253 (41.1) | |
|  | III | 312 (33.1) | 104 (31.6) | 208 (33.8) | |
|  | IV | 13 (1.4) | 5 (1.5) | 8 (1.3) |  |
|  | Unknown | 55 (5.8) | 15 (4.6) | 40 (6.5) |  |
| Laterality (%) | Left | 398 (42.2) | 146 (44.4) | 252 (41.0) | 0.469 |
|  | Other/Unknown | 1 (0.1) | 0 (0.0) | 1 (0.2) |  |
|  | Right | 545 (57.7) | 183 (55.6) | 362 (58.9) | |
| Tumor size (%) | 1-10mm | 45 (4.8) | 9 (2.7) | 36 (5.9) | 0.014 |
|  | 11-20mm | 245 (26.0) | 74 (22.5) | 171 (27.8) | |
|  | 21-30mm | 256 (27.1) | 90 (27.4) | 166 (27.0) | |
|  | 31-50mm | 247 (26.2) | 86 (26.1) | 161 (26.2) | |
|  | 51-70mm | 86 (9.1) | 39 (11.9) | 47 (7.6) |  |
|  | >70mm | 55 (5.8) | 27 (8.2) | 28 (4.6) |  |
|  | Unknown | 10 (1.1) | 4 (1.2) | 6 (1.0) |  |
| M stage (%) | M0 | 914 (96.8) | 317 (96.4) | 597 (97.1) | 0.153 |
|  | M1 | 28 (3.0) | 10 (3.0) | 18 (2.9) |  |
|  | Unknown | 2 (0.2) | 2 (0.6) | 0 (0.0) |  |
| N stage (%) | N0 | 766 (81.1) | 265 (80.5) | 501 (81.5) | 0.986 |
|  | N1 | 90 (9.5) | 32 (9.7) | 58 (9.4) |  |
|  | N2 | 85 (9.0) | 31 (9.4) | 54 (8.8) |  |
|  | N3 | 3 (0.3) | 1 (0.3) | 2 (0.3) |  |
