# Supplementary figures and images for: The clinical-histologic and prognostic characteristics in patients with a second primary non-small-cell lung cancer after a lobectomy
Source: Interdiscip Cardiovasc Thorac Surg. 2023 Sep 15;37(3):ivad155. doi: 10.1093/icvts/ivad155 (PMC10521628; doi:10.1093/icvts/ivad155)

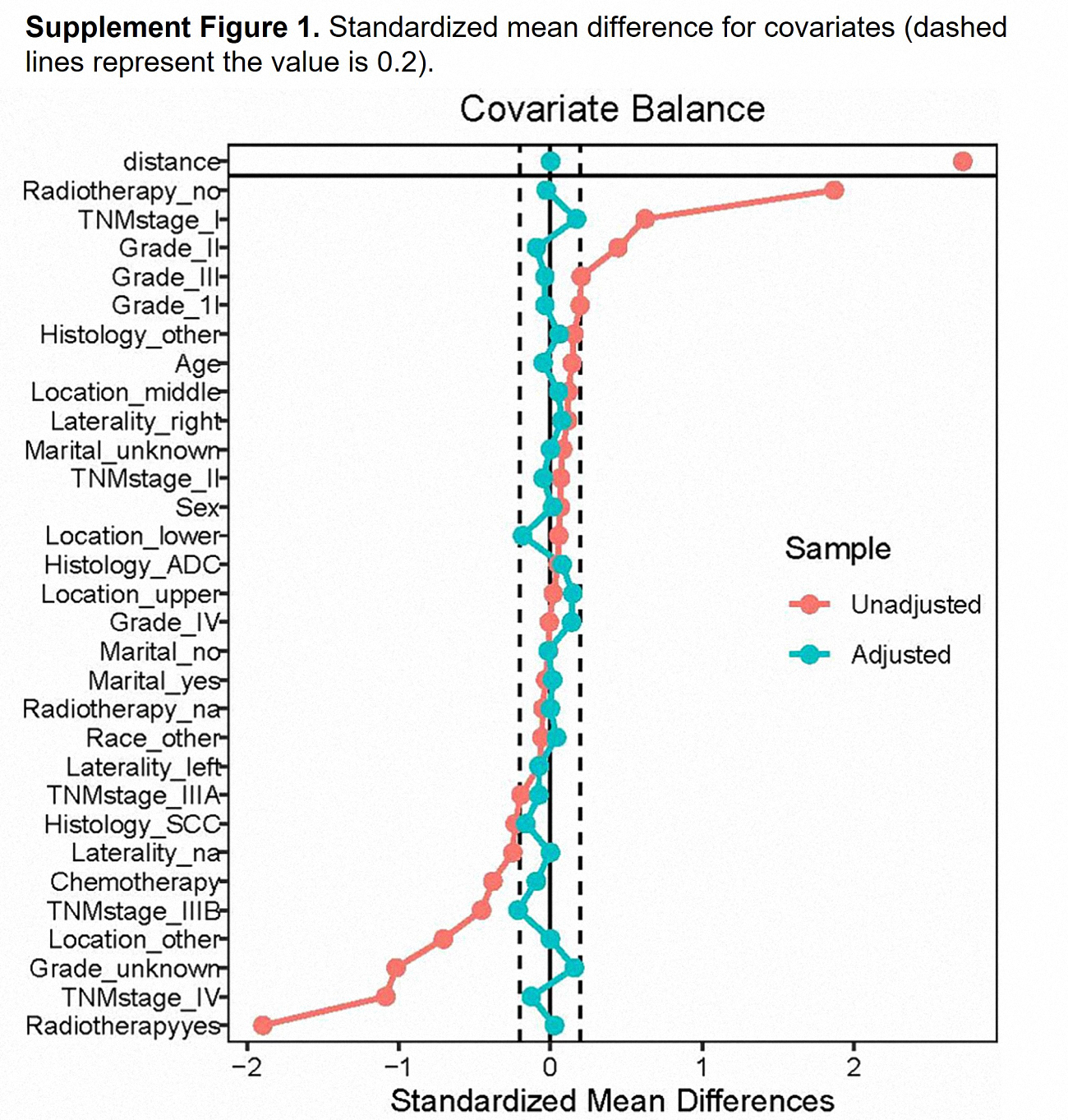

Supplement: ivad155_Supplementary_Data [file ivad155_supplementary_data.zip › Supplementary Figure 1.png]
